# Recommended Best Practices for Lyophilization Validation 2021 Part II: Process Qualification and Continued Process Verification

**DOI:** 10.1208/s12249-021-02107-6

**Published:** 2021-11-08

**Authors:** Feroz Jameel, Alina Alexeenko, Akhilesh Bhambhani, Gregory Sacha, Tong Zhu, Serguei Tchessalov, Puneet Sharma, Ehab Moussa, Lavanya Iyer, Sumit Luthra, Jayasree Srinivasan, Ted Tharp, Joseph Azzarella, Petr Kazarin, Mehfouz Jalal

**Affiliations:** 1 Abbvie, North Chicago, IL USA; 2 Birck Nanotechnology Center, Purdue University, 1205 W State St., West Lafayette, IN 47907 USA; 3 Ultragenyx pharmaceutical Inc., Brisbane, CA USA; 4 Baxter Healthcare, Bloomington, IN USA; 5 Pfizer, Andover, MA USA; 6 Genentech, South San Francisco, CA USA; 7 BMS, New Brunswick, NJ USA; 8 Fresenius Kabi, Buffalo, NY USA

**Keywords:** freeze-drying, lyophilization, process performance qualification (PPQ), continued process verification, heat and mass transfer

## Abstract

This work describes the lyophilization process validation and consists of two parts. Part one (Part I: Process Design and Modeling) focuses on the process design and is described in the previous paper, while the current paper is devoted to process qualification and continued process verification. The goal of the study is to show the cutting edge of lyophilization validation based on the integrated community-based opinion and the industrial perspective. This study presents best practices for batch size determination and includes the effect of batch size on drying time, process parameters selection strategies, and batch size overage to compensate for losses during production. It also includes sampling strategies to demonstrate batch uniformity as well as the use of statistical models to ensure adequate sampling. Based on the LyoHUB member organizations survey, the best practices in determining the number of PPQ runs are developed including the bracketing approach with minimum and maximum loads. Standard practice around CQA and CPP selection is outlined and shows the advantages of using control charts and run charts for process trending and quality control. The case studies demonstrating the validation strategy for monoclonal antibody and the impact of the loading process on the lyophilization cycle and product quality as well as the special case of lyophilization for dual-chamber cartridge system are chosen to illustrate the process validation. The standard practices in the validation of the lyophilization process, special lyophilization processes, and their impact on the validation strategy are discussed.

**Graphical Abstract:**

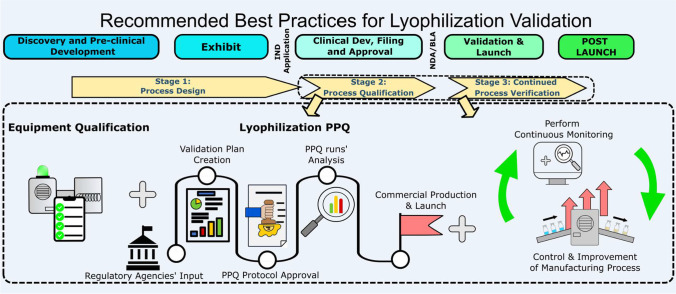

## INTRODUCTION

Lyophilization is a commonly used manufacturing process to prepare dried solid dosage forms of drug molecules that are vulnerable to physical and chemical degradation in the liquid state. The lyophilization process involves three steps; freezing, primary drying, and secondary drying. In the first step, a frozen matrix is formed wherein water is converted into ice crystals leaving behind solutes in the interstitial spaces. In the second step, frozen water is removed by sublimation under vacuum and low-temperature conditions. In the third and final step, the remaining unfrozen water is removed by desorption at relatively high temperatures.

A successful lyophilization process results in lyophilizates that have low residual moisture content and are free from physical defects. In addition, the lyophilization process must be robust over a range of critical process parameters and result in drug products with consistent quality attributes within and across batches. Due to the complex nature of the lyophilization process and the interactions between several product and process parameters, the development, scale-up, and validation of a robust lyophilization process require a thorough understanding of the product and process conditions, as well as the capabilities of the lyophilization equipment.

In general, pharmaceutical process validation protocols are developed following the current United States Food and Drug Administration (US-FDA) guidance to the industry (1). The objective of the lyophilization process validation is the demonstration of the homogeneity of product critical quality attributes (CQAs) within any given batch and their reproducibility across batches to assure that the quality, safety, and efficacy of the drug product is consistent and hence suitable for commercial distribution.

Over the past four decades, the validation of the lyophilization process has been specifically addressed in two reports by Jennings in 1986 (2) and Trappler in 2007 (3). Ever since the publication of the latter report, several advances have been attained in lyophilization technologies, process analytical technology (PAT), computer models of lyophilization process and equipment capability, and the application of the elements of the quality-by-design (QbD) paradigm. Accordingly, an update of the best practices of the validation of lyophilization processes is needed especially given the surge in the number of therapeutic modalities in development pipelines that require lyophilization.

The authors of this paper aim to provide the current perspectives of the pharmaceutical industry on the best practices to meet the expectations of the regulatory authorities on process validation as it pertains to the lyophilization unit operation. The validation of other sterile manufacturing unit operations associated with the freeze-dried product is not the focus of this paper and will only be discussed in cases where the validation of these unit operations are impacted by the validation protocol of the lyophilization process. The discussion in this paper is mainly focused on the lyophilization of aqueous formulations in glass vials, and for completion a case study on dual-chamber syringes will also be shared. Additionally, practical case studies of validation for different therapeutic modalities including therapeutic proteins and vaccines are included for illustration. Moreover, the current perspectives regarding the use of process modeling to support the validation activities are critically discussed along with illustrative examples.

The US-FDA encourages drug manufacturers to use modern pharmaceutical development concepts, quality risk management, and quality systems during the lifecycle of the manufacturing process. Accordingly, in this paper, we take into consideration the current US-FDA guidance to the industry on process validation (1) as well as the International Conference on Harmonization (ICH) guidance for the industry on Pharmaceutical Development (Q8), Quality Risk Management (Q9), and Pharmaceutical Quality System (Q10). Here, however, we do not discuss the validation of automated process control systems (for example, computer hardware and software interfaces), which are commonly integrated into modern drug manufacturing equipment but could be relevant to the validation of processes that include automated equipment.

## LYOPHILIZATION PROCESS VALIDATION

Part I of this best practices’ publication focuses on the early stages of lyophilization product development and process validation with an emphasis on the generation of a design space associated with a given product and equipment. Part II is intended to serve as a continuation of that effort with an emphasis on process qualification, specifically on the qualification of the equipment and well as Process Performance Qualification (PPQ), and Continued Process Verification as currently practiced across various industry partners.

### Stage 1—Process Design

In Part I: Process Design and Modeling (i.e., stage 1), the validation activities for pharmaceutical lyophilization were applied to increase the efficiency of stages 2 and 3. Particularly, the process of product- and equipment-specific design space generation, as well as modeling applications, were demonstrated. The illustrative case-studies are demonstrated to show the value of modeling. The described activities are aimed at improving the process understanding and preparing for Stages 2 and 3 which are described below.

### Stage 2—Process Qualification

After the completion of stage 1, the designed process needs to be evaluated to determine if it is capable of reproducible manufacturing at the commercial scale. As it pertains to lyophilization, stage 2 has two main goals: (1) qualification of the lyophilization equipment including all associated utilities, PAT, and controls; and (2) qualification of the freeze-drying process performance including the loading and unloading processes. This is further described in detail below.

#### Qualification of the Lyophilization Equipment

Qualification of the lyophilizer involves a series of functional tests designed to verify that the utility systems and the equipment operate following the process requirements over all the anticipated operating ranges. This testing is typically done during the installation and operational qualification (IQ/OQ) upon installation and commissioning. Some of the qualification testing relevant to lyophilization process modeling and scale-up (for example, measuring the vial heat transfer coefficient and determining the equipment capability curve) is not usually carried out during IQ/OQ. Such tests can be done before starting the lyophilization process qualification or replaced by values obtained using computational modeling. Also, similar to all multiple-use equipment, cleaning, and sterilization-in-place procedures have to be qualified. Examples of qualification tests for the commercial-scale manufacturing lyophilizer are listed in Table I.

**Table I:** Recommended Qualification Tests for the Commercial-Scale Manufacturing Lyophilizer. These Tests are Executed for Each Lyophilizer in the Manufacturing Lines

Qualification test	Description and target specification
Sensor calibration (Standard IQ/OQ test)	Temperature and pressure sensor calibration Test repeated at specified intervals
Empty chamber temperature mapping: shelf and condenser (Standard IQ/OQ test)	The shelf surface temperature at any spot on one shelf or across shelves is within ± 0.5 °C of the average after equilibration. The cooling rate from ambient to – 45 °C at ≤ 1 °C/min can be achieved The average shelf temperature is within ± 0.5 °C of the set point after equilibration The condenser should reach the setpoint ± 2 °C and maintain that average temperature during the entire run. Condenser temperature is < – 50 °C
Vacuum system (Standard IQ/OQ test)	A pressure of at least 50 mTorr can be achieved from ambient conditions within 1 h and can be maintained within ± 5 units of the setpoint Leak rate meets sterility specification. A frequently specified leak rate for clean, dry, and empty freeze-dryers would be 2 × 10^−2^ mbar-liter/s (15 µm (mTorr) liters/s) (4)
Condenser capacity (Standard IQ/OQ test)	The loss of weight from a weighed amount of water in trays is higher than the stated capacity of the condenser
Stoppering (Standard IQ/OQ test)	All vials are stoppered at the target nitrogen backfill pressure range (250–750 Torr) with no vial breakage or faulty stoppering
Cleaning and sterilization in place cycles (Standard IQ/OQ test)	Repeated at fixed intervals to maintain the validation status of the lyophilizer Certain drug products may require specific cleaning validation procedures
Additional specific tests (Standard IQ/OQ test if capabilities are included)	IQ/OQ of controlled ice nucleation system IQ/OQ of a Tunable Diode Laser Absorption Spectroscopy (TDLAS) system
Hydrocarbon and silicone contamination (Non-standard IQ/OQ test)	Silicone levels on swabs should not exceed the negative control. Hydrocarbon levels on swabs should not exceed the negative control This procedure must be run after a lyophilization cycle. Do not use siliconized stoppers in the lyophilizer
Equipment capability (Non-standard IQ/OQ test)	Minimal controllable chamber pressure at a given sublimation rate (choke condition)
Microbial and Particulate Monitoring Test (Non-standard IQ/OQ test)	Cleaning cycles must be sufficient to prevent cross contamination. Spike and swab locations used during cleaning validation must be justified. All support equipment such as trays, rakes, and forceps must be cleaned and sterilized using validated procedures and handled according to good aseptic practices to prevent their contamination. Soiled, clean, and sterile hold times must be established and qualified Limits/specifications are also applied to these indirect surfaces besides direct product contact surfaces used in filling operations. As an example from our contributors, the shelves can be contaminated with product solution, dried in a simulated cycle, collapsed, cleaned and a swab sample taken from the shelf and above shelf. In addition to removal of potential product residues, a program for cleaning the chamber of broken glass, and lost components must also be defined
Mass Spec, RGA, Filter Integrity Tester (Non-standard IQ/OQ test)	In case of stand alone unit, the IQ, OQ will be done separately or at the same time during the IQ/OQ of the FD. In case of integration to the main controller, these functionalities are usually tested as part of the IQ/OQ testing

#### Lyophilization Process Performance Qualification

The approach to process performance qualification (PPQ) should be based on sound scientific rationale and leverage the cumulative data from all relevant studies to establish the commercial manufacturing lyophilization process. The PPQ batches should involve increased sampling, testing, and scrutiny of the process performance compared to routine commercial batches to confirm consistent product quality within and between batches.

The PPQ runs are conducted according to a written protocol that is typically communicated to the regulatory agencies in advance to seek their input before execution. The protocol should include the validation plan for all manufacturing unit operations that precede and follow lyophilization in the process train. With regard to lyophilization, the protocol should include specific details about batch size, the primary package configuration (vial type and size, stopper type, and fill height), the lyophilization process parameters’ (for example, shelf temperature, drying chamber pressure, and condenser temperature) ranges and/or limits, and the process data to be collected. The protocol should also specify the sampling plan and acceptance criteria for in-process characterization (for example, residual moisture and/or headspace oxygen), and release testing as well as details of the statistical methods to be used for analyzing the process data and the testing samples. Additionally, if computational modeling is employed to justify the number and design of the runs, and the associated testing plans, details of the models used should be included.

Once the protocol is approved internally as well as by the regulatory authorities, the collected data from the PPQ runs are analyzed and a detailed report is written to document protocol execution, data analysis, any process deviations, and non-conformances, and the conclusions of the PPQ. If the results of the PPQ meet all acceptance criteria, the product is cleared for commercial production and launch. Notably, the validation of all unit operations, including but not limited to lyophilization, have to be successful for the drug product to be cleared.

### Stage 3—Continued Process Verification

The third and final stage of process validation is to continuously monitor the performance of the lyophilization process during the manufacturing of the subsequent commercial batches to confirm that the process remains within the validated operating ranges that would yield consistent product quality. This is achieved by establishing an ongoing process to collect and analyze data of the incoming raw materials, the finished drug product, and the process data that are deemed relevant to product quality. The data collection and analysis should be executed following robust statistical methods as well as appropriate qualitative approaches. Additionally, the qualification of the lyophilization equipment should be performed periodically to ensure that the process performance remains within the validated limits. Qualification, or re-qualification, of equipment, is generally only done on a “per cause” basis. For example, calibration checks are done as per performance rather than for continued process verification management. Qualification is performed as part of IQ/OQ and not each time PPQ runs are done. As such, requalifications are done based on the site validation master plan and change management procedures. In cases where qualifications are not done, the rationale should be provided.

Historically, when a sufficient number of batches has been manufactured, process capability can be established; the level and frequency of monitoring and routine sampling of the subsequent batches can be determined. In cases where the data collected during the verification stage suggest that certain changes should be implemented due to changes in the equipment or the drug product, an amendment of the protocol and, if required, additional validation runs should be approved and completed. In certain scenarios, these changes are planned and anticipated at or before the qualification stage. In such cases, change management protocols (5) are typically written and communicated to the regulatory authorities for approval before the introduction of change. Examples of such situations include changes in the fill height/volume and/or vial size and changes in the lyophilization process.

In the following sections of this paper, each of the aforementioned stages of lyophilization process validation is discussed in detail along with the perspectives of the authors on the recommended best practices to ensure successful validation.

## CURRENT PRACTICES IN LYOPHILIZATION PROCESS VALIDATION

PPQ of lyophilization (stage 2 of process validation), like many other processes, has historically relied on the three-drug product lot validation for a given cycle and dose where the validation consists of repeating the process in three consecutive batches of product for a given product image. This is coupled with expanded sampling for critical quality attributes that are most impacted by the lyophilization process, e.g., visual appearance, residual moisture, potency, and reconstitution time. Although the FDA has provided inspection guidance on lyophilization (6), it does not include great detail about PPQ runs, as the guidance predates the current three-stage validation model. To benchmark and understand the current validation practices, Lyophilization Technology Hub (LyoHub), an industry-academia consortium to advance the science and technology of lyophilization, surveyed its industry members that confirmed these practices continue today (Table III).

In the survey of six member companies, 90% answered that they use a standard of 3 maximum load plus 1 minimum load batch for PPQ. Member companies were also asked about any instances where more or less than 3 runs were used for validation. Product families with multiple strengths, fill volumes, etc. typically require more than 3 PPQ batches, but the total number of batches required can be minimized by testing only the representative worst-case configurations. Where multiple lyophilizers are to be used, demonstrated lyophilizer equivalence may be used to allow a minimum of 3 maximum load batches with at least one in each equivalent cabinet plus 1 minimum load batch in any one cabinet. These examples are further exemplified in table format with relevant case studies and survey findings from industry partners (Tables III, IV, and V).

Sampling plans are also an important part of the lyophilized product validation process. The LyoHub member companies were surveyed regarding the types of sampling schemes that are used for lyophilization. The most common sampling plan, at 67%, was the pulling of samples at all 4 corners and from the center of the shelf for each loaded shelf of the lyophilizer. Additional sampling methods included from the top, middle, bottom, and left and right sides of the lyophilizer and may be based on an internal QbD approach. The focus of the sampling plan is to cover a wide range of locations to show product homogeneity across the lyophilizer. The most common critical quality attributes (CQAs) tested by the member companies were visual appearance, residual moisture, potency, and reconstitution time. Cake appearance, may or may not be, a CQA and product performance (e.g., stability, potency) needs to be correlated to appearance to fully appreciate the value of cake collapse.

The last area of focus in the survey covered process modeling and the use of the design space for lyophilization. In general, these areas are of growing interest to the member companies. When asked about the creation and use of a design space for lyophilization, 80% of member companies use it, but only one company has submitted a filing to the US-FDA that has included the design space. The next section of this best practice paper will focus in detail on lyophilization process validation and ways that modeling can be used to support the validation. As the design space and models used to create them have become more accepted, the models can also provide ways to minimize the number of validation runs. Given the diversity of approaches used across the industry, the authors decided to compile a white paper that provides a harmonized recommendation for best practices as well as a future outlook for the use of modeling.

## STAGE 2—PROCESS PERFORMANCE QUALIFICATION

PPQ is the second part of stage 2 of process validation. According to *FDA Guidance 2011, Process Validation: General Principles and Practice* (5), the goal of PPQ is to demonstrate that the commercial manufacturing process performs as expected and confirms process design through the use of the following five elements (i) qualified facility and utilities, (ii) equipment and components (iii) trained personnel, (iv) well-understood manufacturing process, and (v) control procedures (5). Successful completion of PPQs is required before commencing commercial production of the drug product batches. The approach for PPQ should be based on manufacturers’ sound scientific understanding of the product and manufacturing process and its relation to the combined use of the five elements mentioned above. Manufacturing conditions during PPQs are often based on an understanding of the qualified scale down process models and the cumulative data generated during clinical manufacturing and small scale, pilot, and commercial-scale studies. It is expected that PPQ will involve extensive sampling, additional testing, and higher scrutiny to ensure homogeneity in drug product quality throughout the batch. The duration to continue extensive sampling and additional testing should be based on a continued process verification program which includes considerations for the volume of production, process complexity, understanding of the process, and experience with similar products and processes (5).

The following prerequisites are typically completed before the execution of a PPQ study:

- Identification of the glass transition temperature of the frozen matrix (T_g_’) and collapse temperature or critical temperature which is an indication of the product failure point
- Facility design/equipment/automation/utility installation, operational, and performance qualification, where applicable
- Drug substance (API) validation (for drug product validation)
- Rationale documented for selection of critical quality attributes (CQAs) and critical process parameters (CPPs), and their respective specifications and process parameter ranges
- Aseptic process qualification/validation (simulation challenges, if applicable)
- Sterilization/depyrogenation validation (if applicable)
- Validation of sterilizing filters (if applicable)
- Container closure integrity validation (if applicable)
- Analytical methods validation/verification/qualification (including PAT methods)
- Data analysis report containing an assessment of development/historical process data to support the selection of acceptance criteria for statistically-based sampling plans
- Standard operating procedures (SOPs) required for process validation written and approved
- Preventive maintenance (PM) program implemented
- Training (GMP and process specific)
- Approved PPQ protocol containing pre-defined acceptance criteria
- Equipment verified clean before processing

It should be noted that cleaning validation may be performed concurrently with PPQ and the list above is not meant to be prescriptive. Before returning the equipment to commercial processing after the completion of PPQ, however, either cleaning validation must be completed and approved or a successful cleaning verification must be performed.

### PPQ Protocol

The protocol for lyophilized products has specific elements to assure that the manufacturing process will consistently produce a drug product that meets all predefined acceptance criteria.

#### Batch Size

Minimum and maximum batch sizes for lyophilized products are defined in terms of the shelf load. For example, for a lyophilizer with 10 shelves, a minimum batch size for one specific drug product configuration could be 1 shelf load and the maximum batch size could be the 10 shelf load. Note that the maximum batch size for compounding and lyophilizer can be different.

PPQ protocol should include the type and number of vials or units to be loaded for the minimum and maximum batch size. Depending on the production volume, it may be acceptable to use one partially filled shelf as the minimum load (especially for low turnover products) provided that the product quality of the batch is supported by appropriate commercial-scale studies and manufacturing controls.

The following are some considerations to take while determining batch sizes:

- In terms of the lyophilization cycle, partial or small batch sizes are expected to complete the drying faster than bigger batch sizes (7).
- The upper range of lyophilization cycle parameters for sublimation (i.e., shelf temperature, chamber pressure) should be set to avoid product failure.
- When deciding on the size of the small batch, care should be taken to account for filling line losses, in process, and release samples (i.e., dead volume, sampling plan, and release criteria).
- Lyophilizers must be capable of maintaining pressure within the acceptable range. The maximum batch size presents the worst case for sublimation and condenser capacity with the amplified center to edge vial Kv differences. Therefore, lyophilization cycle parameters should be carefully selected and/or verified to generate a vapor flux that can be supported by the lyophilizers at the maximum batch size. Additionally, the maximum batch size should be selected such that total net water to be removed is always below condenser capacity (quantity of water in the form of ice that can be deposited on the condenser surface).
- Allocated liquid hold time should allow for maximum batch size to be filled and loaded in the lyophilizer. This is especially true for vaccines wherein Time in Solution (TIS) is a critical process parameter and potency can be lost per hour while waiting to load the full cabinet. Similar would be the case for an antibody program where phase separation is observed over time and thus worst-case TIS should be used for consistent product performance.
- Location of shelf for small batch is also important. For instance, if the validation is done on the top shelf, subsequent commercial batches should be the same shelf.

#### Number of PPQ Lots

A pre-defined number of PPQ lots are manufactured to generate a robust understanding of the variability in the manufacturing process. Data from the PPQ batches must provide a high degree of assurance that the manufacturing process is reproducible, implementation of the control strategy is robust, and hence support the commercial release of the drug product. Determination of the number of lots to manufacture depends on many factors. For example, the complexity of the manufacturing process and product, variability in the manufacturing process, process understanding gained during scale down and commercial-scale studies, and overall experience of the manufacturer with the manufacturing process.

Three batches at maximum lyophilizer load and one batch at minimum lyophilizer load were suggested during the CASS CMC Strategy forum in 2016 (8). However, with appropriate justification based on a sound manufacturing control strategy, it is acceptable to use a different approach for the number of PPQ batches. Tables III, IV, and V present the results of a survey for the number of PPQ batches used by six pharmaceutical companies for lyophilized products.

In cases where two dose strengths and more than one lyophilizer are in the scope of PPQ, a bracketing approach can be used to minimize the number of PPQ runs. It is recommended that appropriate studies are in place to demonstrate the functional equivalency of lyophilizers to minimize the number of PPQ runs by being able to use the lyophilizers interchangeably between the PPQs and also for commercial manufacturing flexibility.

Below are the results of a survey for the number of PPQ runs at maximum and minimum lyophilizer loads used by various pharmaceutical companies for four cases (Table II).

**Table II:** Cases Considered for Survey of Number of PPQ Runs at Maximum and Minimum Lyophilizer Loads. Survey Results for Cases A, B—Table III; C, D—Table IV; E, F—Table V

	Case A (Table III)	Case B (Table III)	Case C Table IV	Case D Table IV	Case E Table V	Case F Table V
Number of presentations	1	1	1	1 but different vial suppliers	2	2
Number of lyophilizers	1	2 equivalent lyophilizers	2 or more non-equivivalent lyophilizers	1	1	2

**Table III:** Survey Findings Associated with the Number of PPQ Runs at Maximum and Minimum Lyophilizer Loads Used by Pharmaceutical Companies for Case A. Assumption: Drug Product will be Filed with the US FDA. The Survey Question as Asked to the Industry Leads is Listed Here. How Many Runs Would you Typically do for Each of the Following Scenarios? Assume the Same Formulation Composition in all Scenarios. What Lyo Load Would You Use for These Runs for a Low or a High Volume Product?

	Company A	Company B	Company C	Company D	Company E	Company F
Case A: 1 presentation (vial/fill) and 1 lyophilizer	3 max, 1 min; 2 max, 1 min; in rare cases 1 max, 2 min	3 runs total	3–5 runs total	3 max, 1 min	3 runs spanning max and min	3 max, 1 min
Case B: 1 presentation (vial/fill) and 2 or more equivalent lyophilizers	1 max, 1 min per dryer	3 runs; 1 run per dryer up to three dryers	3–5 runs total A batch does not need to be made in each lyophilizer if they are equivalent	2 max, 1 min run on dryer 1; 1 max on dryer 2	3 total PPQ runs using both lyophilizers spanning max and min loads	2 max, 1 min run on dryer 1; 1 max on Dryer 2

**Table IV:** Survey Findings for Number of PPQ Runs for Maximum and Minimum Lyophilizer Loads Used by Pharmaceutical Companies for Case B. Assumptions: (i) Large Batch Size Requiring Either Mulitple Lyophilizers to be Loaded or Different Vial Supplier for Commercial Manufacturing. (ii) Drug Product will be Filed with US FDA. Survey Question as Asked to the Industry Leads is Listed Here: “How Many Runs Would You Typically do for Each of the Following Scenarios? Assume the Same Formulation Composition in all Scenarios. What Lyo Load Would You Use for These Runs for a Low or a High Volume Product?”

	Company A	Company B	Company C	Company D	Company E	Company F
Case C: 1 presentation (vial/fill) and 2 or more non-equivalent lyophilizers	2 max, 2 min on each dryers	3 runs per dryer	3–5 runs A minimum of 1 batch is required in each lyophlizer	3 max, 1 min on each dryers	3 runs spanning max and min per dryer	3 max, 1 min on each dryers
Case D: 1 presentation (vial size/fill) and different vials suppliers (ex. Schott vs. OMPI), 1 lyophilizer	No real-life experience; hypothetically 2 max, 2 min per image	4 to 6 runs, depending on vial differences AND if dryers are the same and batch size are the same	3–5 runs per image; A minimum of 3 batches are required in each lyophlizer	3 max, 1 min per image Specification and risk based approach	3 runs spanning max and min per image	3 max, 1 min for each image if different Kv If Kv is same, vials are deemed equivalent and not part of validation

**Table V:** Survey Findings on the Number of PPQ Runs for Maximum and Minimum Lyophilizer Loads Used by Pharmaceutical Companies for Case 2. Assumption: 2 Different Presentations with *One* or Two Lyophilizers to be loaded for commercial manufacturing. Assumption: Drug Product will be Filed with the US FDA. The Survey Question as Asked to the Industry Leads is Listed Here: “How Many Runs Would You Typically do for Each of the Following Scenarios? Assume the Same Formulation Composition in all Scenarios. What Lyo Load Would You Use for These Runs for a Low or a High Volume Product?”

	Company A	Company B	Company C	Company D	Company E	Company F
Case E: 2 presentations (2 different fill volumes in the same vial or 2 different fill volumes in two different vials) and 1 lyophilizer	Independent validation required for each presentation (i.e., vials or fill volume) as they would have different lyocycle	6 total, 3 per presentation	3–5 runs per presentation; A minimum of 1 batch is required for each presentation	3 max, 1 min for each presentation assuming cycles are different	Separate PPQs for each presentation. For two different fill volumes, potential to reduce number of PPQs	3 max, 1 min of each fill volume (no min for small size batch) for each dose strength
Case F: 2 presentations (2 different fill volumes in the same vial or 2 different fill volumes in two different vials) and 2 or more lyophilizers	Combination of response in cases B, C, and E	6 total, 3 per presentation; at least one per dryer if equivalent—more if not	Combination of response in cases B, C, and E	Combination of response in cases B, C, and E	Combination of response in cases B, C, and E	3 max (2 on one lyophilizer and 1 on every other equivalent lyophilizer) for each dose strength. 1 min only for large size batch

Generally speaking, there was a consensus across industry leads on the conservative definition of lyophilizer equivalency. The same manufacturer, model, and size, for example, were unanimously considered as equivalent. Units from the same manufacturer with a matching model size but different manufacturing dates were considered equivalent only after performing, comparable equipment capability curve with Kv value and temperature mapping comparison. However, a thorough discussion prompted the need to define the equivalency of lyophilization cabinets based on mechanical equivalency and process equivalency. Mechanical equivalency can be determined by equipment validations factors such as minimum/maximum shelf temperature, maximum sublimation rate, minimum controllable pressure, and condenser temperature while process equivalency is determined by making batches of the same product in each lyophilizer and performing statistical analysis of the product attributes in both lyophilizers (assay, water content, etc.) to see if product generated in both lyophilizers is equivalent.

Cases E and F (Table II), for example, may provide the number of PPQ runs for two dose strengths. As mentioned above, the bracketing or matrixing approach is acceptable to minimize PPQ runs when applicable. To apply bracketing, one approach may rely on categorizing the dose strengths into high risk (HR) and low risk (LR) for impact by the lyophilization unit operation at maximum load and then determine the total number of PPQ runs to bracket LR dose strength. These differences between the dose strengths need to be taken into consideration for risk categorization. As an example, for lyophilized formulations containing protein and sugar, a change in protein concentration may also necessitate a change in protein to sugar ratio, a change in the fill volume may necessitate a change in the vial size, and these changes may require the use of different lyophilization cycles.

Tables VI and VII below provide an example of an approach to categorize dose strengths as high risk for model drug product configuration with low and high protein: sugar ratio. It is assumed that the same lyophilization cycle is used for both dose strengths.

**Table VI:** Illustrative Examples of Product Presentations Showcasing a Model Drug Product Configuration with Low Protein: Sugar Ratio in Low Dose and High Dose Strengths

Drug product configuration	1		2		3	
	Low dose	High dose	Low dose	High dose	Low dose	High dose
Dose (mg/vial)	5	10	5	10	5	10
Protein concentration (mg/mL)	5	5	5	5	5	10
Fill volume (mL)	1	2	1	2	1	1
Vial size (cc)	5	5	2	5	5	5
High risk	High dose		Low dose		High dose	
Rationale for worst case (link rationale to impact to a CQA, shelf life or process/equipment capability to meet process ranges)	Higher fill volume and thus high total solid content may result in high variability in moisture and long drying time If the process parameters are aggressive for the product, slight fluctuations in process control may impact product quality		Small configuration may show more variability in moisture as a function of hot and cold spots on shelf and higher fill volume/ internal Surface area ratio *(High dose could be worst case if condenser capacity is limited)		High total solid content may result in high variability in moisture If the process parameters are aggressive for the product, slight fluctuations in process control may impact product quality	

**Table VII:** Illustrative Examples of Product Presentations Showcasing a Model Drug Product Configuration with High Protein: Sugar Ratio in Low Dose and High Dose Strengths

Drug product configuration	1		2		3	
	Low dose	High dose	Low dose	High dose	Low dose	High dose
Dose (mg/vial)	250	500	250	500	250	500
Protein concentration (mg/mL)	50	50	50	50	50	100
Fill volume (mL)	5	10	5	10	5	5
Vial size (cc)	20	20	10	20	20	20
High risk	High dose		High dose		High dose	
Rationale for worst case (link rationale to impact to a CQA, shelf life or equipment capability to meet process ranges)	Higher fill volume and high total solid content may result in high variability in moisture If the process parameters are aggressive for the product, slight fluctuations in process control may impact product quality		High dose could be worst case if condenser capacity is limited		Higher total solid content may result in high variability in moisture If the process parameters are aggressive for the product, slight fluctuations in process control may impact product quality	

#### Potential CPPs to be Monitored

A PPQ protocol includes set points and ranges for process parameters’ alarm limits for the lyophilization cycle. PPQs are run at ‘target’ process parameters, which along with their ranges are defined based on scale down or commercial scale studies conducted before PPQ as defined in the section on process design studies. Normal operating ranges for lyophilization cycle process parameters are always within the process parameter ranges.

Process parameters relevant to the lyophilization cycle recipe are as follows:

- Freezing—shelf temperature
- Primary drying—shelf temperature and chamber pressure
- Secondary drying—shelf temperature and chamber pressure

Additional process parameters recommended for process monitoring of the lyophilization cycle include Pirani pressure, nitrogen bleed rate, condenser temperature, and condenser pressure. A typical lyophilization recipe is provided in Table VIII.

**Table VIII:** Sample Lyophilization Process Recipe

Process	Shelf temperature	Chamber pressure	Duration
Loading	X	–	X
Freezing set point	X	–	X
Freezing hold	X		X
Evacuation	X	X	-
Primary drying set point	X	X	X
Primary drying hold	X	X	X
Secondary drying set point	X	X	X
Secondary drying hold	X	X	X
Pre aeration	X	X	–
Stoppering	X	X	–
Aeration	X	X	–
Storage	X	–	–

A value for process parameter is entered in the boxes containing a cross (X). Additional parameters related to stoppering step include stoppering pressure and stoppering hold time (contact time for top of the stopper surface and shelf surface after achieving stoppering pressure set point)

#### Potential CQAs to be Tested

In addition to the CQAs related to the physicochemical and biological properties of the drug product, CQAs specific to the lyophilized product such as cake appearance, residual moisture, and reconstitution time is also part of the release control system. The PPQ protocol should include the rationale for the inclusion of each CQA and sampling frequency. For example, deviation in process parameters, such as shelf temperature, chamber pressure, and primary drying time, outside of the acceptable ranges during a lyophilization cycle can impact cake appearance, residual moisture, and reconstitution time, and hence these attributes are assessed during process validation. To demonstrate uniformity in drug product quality attributes throughout the batch of vials, extensive sampling is performed for PPQ batches. Residual moisture is one attribute that is tested more extensively than the rest of the CQAs. To demonstrate drying uniformity throughout the lyophilizer(s), samples are collected from various locations on the shelves. The selection of shelf locations and the number of samples collected from each location should be based on prior knowledge of variability in shelf surface temperature and moisture. The locations selected for sample collections should be the worst cases in terms of impact on moisture content. Shelf surface temperature variability relative to hot and cold shelf temperature setpoints should be measured during initial equipment qualification and periodic maintenance. Data from these studies serve to identify worst-case locations. Additionally, the impact of shelf surface temperature variability on moisture content can be determined by conducting lyophilization runs using an appropriate surrogate lyophilized product. Together, these studies help in the identification of worst-case locations with hot and cold temperatures on the surface of the shelves. Typically, four corners and the center of each shelf are used as sampling locations since heat transfer to the product is expected to be lowest in the center (cold vials) and highest on the corners which may cause variability in the moisture results. It is recommended that a sampling plan for testing residual moisture be based on relevant statistics to be able to evaluate variability among different locations and the probability of exceeding lot release specification. If a particular location(s) is known to be more variable in shelf surface temperature, this information should be used in the statistical model to determine the appropriate number of samples. Testing of all CQAs from the top, middle, and bottom shelves is recommended to demonstrate drying uniformity in the lyophilizer. Table IX summarizes the recommended product critical quality attributes to be tested in each of the PPQ batches of an exemplary protein product.

The number of samples used for testing should be more than what is required to complete all the testing and to provide for any retests. Additionally, the number of data points (replicates) for each test should be adequate to provide quantitative evidence of inter and intra batch variability. The use of qualified high throughput analytical methods such as Near Infrared (NIR) spectroscopy for moisture testing is acceptable for testing validation samples. A bridging study is required between the high throughput analytical method and lot release analytical method used for testing of validation samples and lot release samples, respectively, to be able to use the validation samples to rationalize sampling plan for lot release testing and specification acceptance criteria (9) (Table IX).

**Table IX:** Recommended Product Critical Quality Attributes (pCQAs) to be Tested in Each of the PPQ Batches of an Exemplary Protein Product

pCQA		Assay/method	Sampling locations in the lyophilizer	Acceptance criteria*
Lyophilized drug product				
Appearance (color, height, shrinkage, cracks, collapse)		Compendial (USP 1 General Chapter on Injections and Implanted Drug Products)	Center and four corners of the top, middle, and bottom shelves	White to off white (or product-specific specification) with no or minimal signs of collapse and cracks
Moisture content		Karl Fisher (USP 921 General Chapter on Water Determination)		≤ 1% (or product-specific specification)
Reconstitution time		Compendial (USP 1 General Chapter on Injections and Implanted Drug Products)		≤ 5 min
Reconstituted drug product				
Appearance	Appearance		Center and four corners of the top, middle, and bottom shelves wherever possible or Beginning, middle, and end of filing (loading/unloading of the lyophilizer)	Practically free from particles
	Color			Product-specific
	Clarity			Product-specific
Identity	HPLC Cytotoxicity			Comparable to standard or ELISA
Protein content	UV–Vis			+ 10% of target concentration
Excipients Example: (Polysorbate 80)	HPLC			+ 0.005% of target concentration
Potency	Bioassay			Report value
Sub-visible particles	Light obscuration or membrane method (where applicable)			particles ≥ 10 µm: ≤ 6000 per container particles ≥ 25 µm: ≤ 600 per container
Visible particles	Compendial (USP 34 General Chapter 788 on Particulates in Injection.)			Practically free from particles
General	Osmolality			+ 50 mOsm/kg of target value
	pH			+ 0.5 units of target value
Safety	Endotoxins			≤ 5 EU
	Sterility			No microbial growth
Purity (main, HMW, and LMW peaks)	SEC-HPLC			Main peak: ≥ 95.0%; Aggregates: ≤ 3.0%
Purity (main peak, acidic, and basic peaks)	CEX-HPLC iCIEF			Main peak: product-specific Report acidic and basic peaks Comparable to reference standard

*No statistically significant difference between samples in each location and across samples from different locations on the same shelf and between shelves. Some of the acceptance criteria are product specific. An appropriate sample size is selected based on prior knowledge of variability to enable appropriate study power.

## STAGE 3—CONTINUED PROCESS VERIFICATION

Continued process verification is categorized as stage 3 of process validation. Manufacturing firms are required to establish and maintain a continuous monitoring program to, at a minimum, annually report the process and product quality data (5). The purpose of this program is to assure that the manufacturing process is in a state of control throughout the lifecycle of the product. The data and information generated during the program also form the basis for identifying improvements to the manufacturing process.

In this lifecycle management state, a continued verification program links the operational elements of the quality system, such as annual product review and change control, to continuous improvement initiatives for the manufacturing process. Fundamental to achieving these goals is the selection and trending of process parameters and quality attributes concerning their specifications. Control and run charts using historical data are used for depicting any atypical and unexpected shifts and patterns in the process parameters and quality attributes over time. The atypical or special cause variation is different from common cause variation in that special cause variation in the process can be attributed to an assignable cause which can be removed through corrective actions leading to process improvement. A well-established set of rules (such as Nelson rules (10) or Western Electric rules (11)) can be used to detect patterns in the process monitoring data and indicate special cause variation.

### Use of Run or Control Chart

A run chart shows a general trend of a process parameter over time. For example, for chamber pressure, it can be the data collected every minute over the different stages of the lyophilization cycle. For multiple batches manufactured in a year, a run chart can be constructed by plotting maximum and minimum values of the chamber pressure for each batch. Different options for plotting a run chart are provided in the section below.

A control chart is a statistical quality control tool to show process stability—average and variability in a process parameter. Variability is expressed in terms of upper control limits (UCL) and lower control limits (LCL). Typically, UCL and LCL are calculated as average ± 3 × standard deviation and reflect process stability. In a perfectly stable process, assuming data variation is normally distributed (random, uncontrolled variation inherent to the process—common cause variation), 68% of the data should fall within 1 standard deviation, 95% of the plotted data should fall within 1.96 standard deviations, and 99.73% of the data should fall within 3 standard deviations (12). Control charts may also have specifications for quality attributes and process parameters (upper specification limit (USL) and lower specification limit (LSL)) which are wider than UCL and LCL. Specifications for quality attributes are derived during stages 1 and 2 of drug product process validation wherein the impact of critical material attributes (CMAs) and process parameters on quality attributes is established. Understanding the impact of CMAs and process parameters on quality attributes together with the safety and efficacy data from clinical studies is used to establish specifications for quality attributes.

**Fig. 1:**
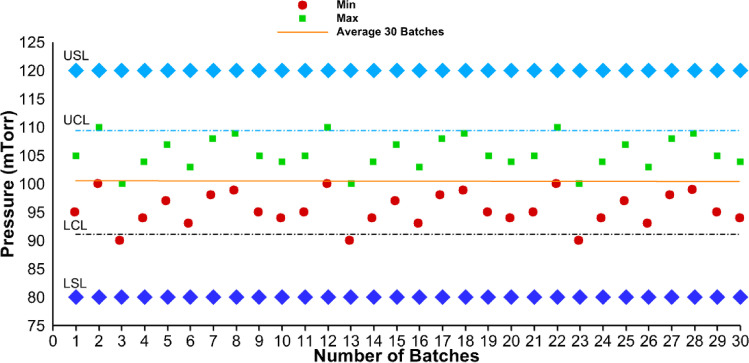
HypotheticalFi trend chart for chamber pressure for 30 drug product batches. Legend: Min = minimum chamber pressure for each batch; Max = maximum chamber pressure for each batch; UCL = upper control limit; LCL = lower control limit; USL = upper specification limit; LSL = lower specification limit

Figure 1 depicts a hypothetical example of a trend chart for minimum and maximum chamber pressures for each of the 30 batches of a drug product. As a worst case, 0–10% variability relative to a set point pressure of 100 mTorr was randomly applied to each of the 30 batches. UCL and LCL were calculated using average ± 3 × standard deviation in pressure data for 30 batches. Note that the USL and LSL of 120 and 80 mTorr are acceptable process parameter limits derived from lyo process robustness studies performed during stage 1—process design of the process validation. For demonstration purposes, pressure data for batches 2 and 3 were shown to be slightly outside the UCL and LCL, respectively.

**Fig. 2:**
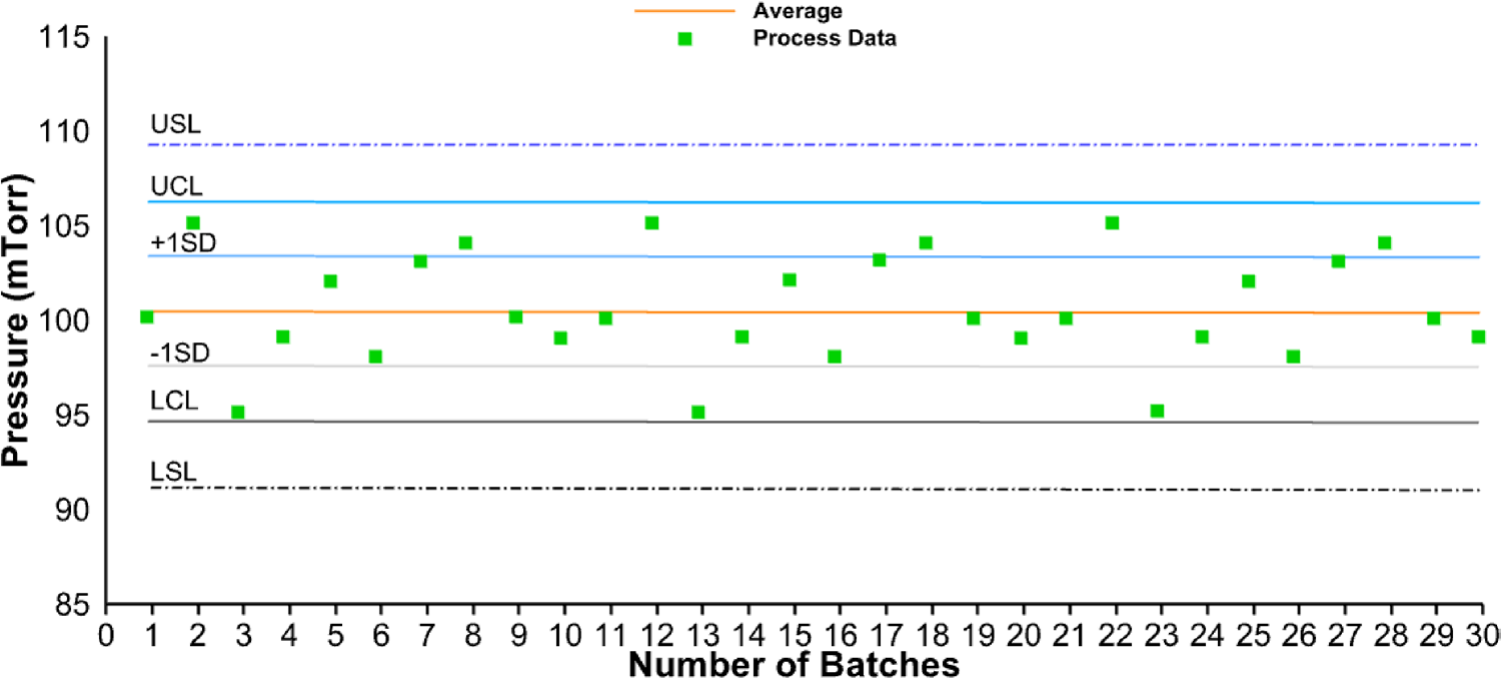
Hypothetical control chart for average chamber pressure for 30 drug product batches. Legend: UCL = upper control limit; LCL = lower control limit; USL = upper specification limit; LSL = lower specification limit; SD = standard deviation

To identify whether there is a shift or pattern in the data, a control chart was constructed (Fig. 2). Here, average pressure data was plotted for 30 batches, and variation in the data was compared against the Nelson rules to identify shifts or patterns in the data. Variation in the data does not fall into any of the rules and therefore can be classified as common cause variation and corrective action is not needed.

### Selection of CQAs and CPPs to Use in the Plot

#### Lyophilized Products in Vials

Quality attributes including in-process controls and release testing of a drug product batch are in the scope of a continued verification program. It is expected that the criticality of these quality attributes is decided based on a scientifically sound control strategy as outlined in ICH Q11 following the principles of Quality Risk Management (ICH Q8). As part of developing a control strategy, it is also important to monitor changes in process inputs including excipients and container closure systems. For example, variation in glass vial dimensions may impact heat transfer to the product during lyophilization.

Quality attributes unique to the lyophilized products in vials are reconstitution time, residual moisture, headspace pressure, and lyophilized cake appearance. While reconstitution time, residual moisture, and headspace pressure are reported out as numeric values and hence are straightforward to use for statistical analysis using control charts, report out of cake appearance being descriptive cannot be used directly for statistical analysis. Percentage reject or more specifically percentage of cake defects per batch may alternatively be used for statistical process verification.

Another factor that does not negatively impact the deliverable dose and critical quality attributes of the product in vials but do impact the the aesthetic/product elegance and to some extent the total yield is the presence of spots and streaks. Spots and streaks are referred to a phenomenon where the product is deposited on the body or on the shoulder of the vial. It is believed to occur due to product solution splashing during filling process which does not drip down into bulk solution but stays and gets frozen during freezing process and get lyophilized leaving behind white streaks and spots. Some of the corrective and preventive actions (CAPA) that can be applied to address these issues include optimization of the filling speed, the nozzle size and the line speed to minimize the rocking of the vials on lines feeding into lyophilizer. A similar phenomenon called fogging is also seen commonly which is attributed to Marangoni flow where the liquid slowly rises even after carefully filling which gets lyophilized and leaves behind a white layer or mist of powder. It is believed a hydrophobic coating of silicon oil inside the vial mitigates the problem (13). In some cases the product solution can form a ring around the neck/shoulder which is referred to “Halos” during drawback of the needle in the filling process and is attributed to both filling speed and the static charges developed on the vial during the vial washing, drying, and depyrogenation steps of manufacturing. In such situations, it can impact the deliverable dose if it does not get reconstituted with the normal reconstitution procedure/method and may require inverting the vial during reconstitution to get that powder reconstituted. If the product is found in the stopper area, then this could raise concern on container closure integrity (CCI) and could potentially be classified as reject.

Shelf temperature, chamber pressure, and time are lyophilization-specific critical process parameters having defined setpoints for different stages of the lyophilization process (freezing ramp rate, freezing hold time, annealing ramp rate and hold time, primary and secondary drying shelf temperature ramp rates and hold times, chamber pressure setpoint during primary and secondary drying). Depending on the specifications established for the process parameter for a given lyophilization stage and on the capability of the equipment to control the parameter within the specifications, not all process parameters may be categorized as critical process parameters and may not be included in the process monitoring program by default. Regardless, as a best practice, it is recommended to periodically monitor all aforementioned lyo cycle parameters.

Tests and checks performed during preventive maintenance of the lyophilizer should also be part of a continued verification program. Results from tests including empty chamber shelf mapping, leak rate with and without closing the isolation valve (if present), capability for fast and slow ramping of temperatures and pressures in the chamber and condenser, and control at minimum pressure are very valuable to monitor.

### Options for Plotting Variations in Control Chart

#### Sample

Variation in a process parameter for a single batch or multiple batches can be plotted using three options (14):

1. Parameter variation: using this option, process parameter values for each process step are plotted in a chart. For trend analysis of multiple batches, this plotting technique will yield a complicated graph which can be difficult to analyze.
2. Deviation from target: using this option, process data is plotted as a deviation from the set point for each process step. Similar to the previous option, this plotting technique will yield complicated graphs when multiple batches are included.
3. Greatest variation using standard deviation or *z*-score: for a large number of batches, plotting all process data as standard deviation simplifies the construction and analysis of the data (Table X).

**Table X:** Considerations for the Three Methods for Plotting Control Charts Mentioned Above

Parameter variation	Deviation from target	Greatest variation using the standard deviation or *z*-score
Plot parameter values for temperature and pressure	Plot the difference in parameter values from target	Use statistical output to plot
Plot each step	Plot each step	Greatest variation throughout the process
Complicated charting each step, difficult for analysis	Complicated charting each step, difficult for analysis	Easier to trend
Overly sensitive to variation	Extremely sensitive to variation	Less sensitive to variation

### Options for Plotting Data in Run Chart

#### Sample

**Fig. 3:**
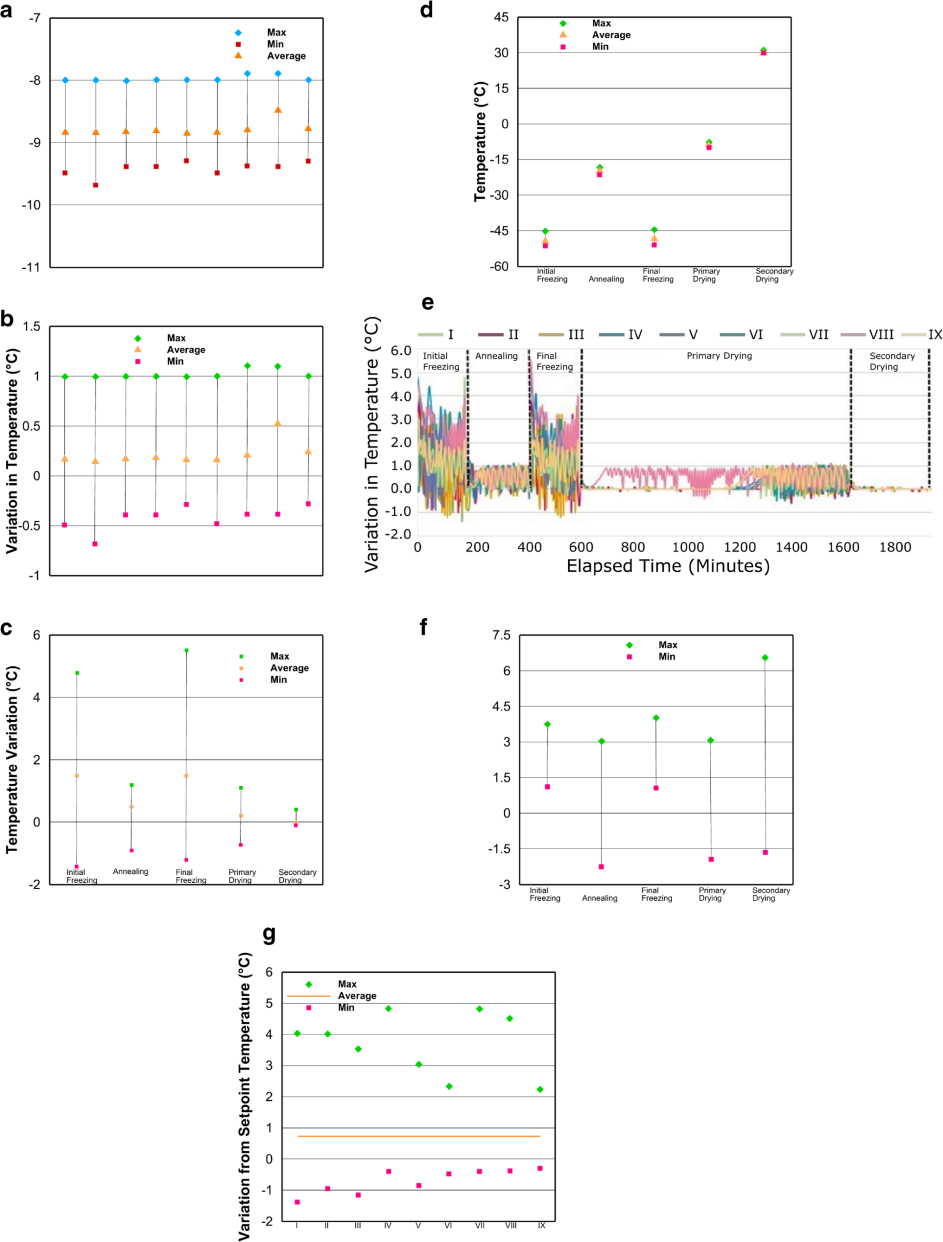
Run charts plotted using different methods listed in Table XI. **a** Method A. Average, max, and min values of shelf temperature for one step of the lyophilization cycle (primary drying) are plotted for 9 batches. **b** Method B. Average, max, and min values of variation in shelf temperature from the set point for one step of the lyophilization cycle (primary drying) are plotted for 9 batches. **c** Method C. Average, max, and min values of variation in shelf temperature from setpoint for all steps of the lyophilization cycle are plotted for 9 batches. **d** Method D. Average, max, and min values of variation in shelf temperature from setpoint for all steps of the lyophilization cycle are plotted for one batch. **e** Method E. Average, max, and min values of actual shelf temperature for all steps of the lyophilization cycle are plotted for 9 batches. **f** Method F.The standard deviation from the mean for shelf temperature for all steps of the lyophilization cycle are plotted for a single batch. **g** Method G. Overall variation from running average in shelf temperature for all steps of the lyophilization cycle are plotted for 9 batches

Table XI  and Fig. 3 present various options for plotting data for run charts. Methods A and E are options where process parameter values are used for plotting run charts. Methods B, C, and D are options where deviation from target or setpoint values is used for plotting. Methods F and G are options where a statistical output, such as standard deviation or range, is used for plotting the data. The decision of which options to choose for plotting largely depends on process characteristics, subgroup data size and data sampling frequency, need and sensitivity to detect small shifts in the process, and goal of the data analysis (14).

**Table XI:** Methods to Plot Run Charts

Methods	Method description	Values to plot	Each/all process step	Number of batches to plot	Corresponding figure
Method A	Actual shelf temperature	Average, max, and min	Each	All batches	a
Method B	Variation from set point	Average, max, and min	Each	All batches	b
Method C	Variation from set point	Average, max, and min	All	All batches	c
Method D	Variation from set point	Average, max, and min	All	Single batch	d
Method E	Actual shelf temperature	Average, max, and min	All	All batches	e
Method F	Standard deviation from the mean shelf temperature	Max and min	All	Single batch	f
Method G	Overall variation from running average for shelf temperature	Max and min	All	Each batch	g

## SPECIAL CASES OF LYOPHILIZATION PROCESS VALIDATION

### Validation Approaches to Freeze-drying of Pharmaceuticals in Alternative Containers

#### Emerging and Existing Container Closure Systems

Dual-chamber containers (vials (DCVs), syringes (DCSs), and cartridges (DCCs)) are emerging container closure systems developed to improve patient convenience during administration. The benefits of dual-chamber systems also include increased patient safety (decreased number of dose preparation steps), decreased dose administration errors (reduction of admixture loss during reconstitution), and cost reduction due to no need for separate diluent containers (15).

##### Dual Chamber Vials

A dual-chamber vial (Act-O-Vial, as an example (15)) comprises of two compartments: the lower compartment contains lyophilized product while the upper compartment is filled with the diluent. Compartments are separated by an elastomeric stopper. The product is filled and lyophilized followed by stopper placement and diluent fill operations. Due to the configuration, the container cannot be stoppered as they typically are within the lyophilizer; the lyophilized product is exposed to environmental conditions before stoppering. It is therefore critical to maintain low relative humidity and controls to prevent microbial contamination of the environment in which the product will be stoppered. To administer the content of a DCV, the user needs to press a plastic activator to push the stopper down to the lower compartment allowing the diluent to come in contact with the lyophilized powder. To make sure that the stopper moves smoothly, after the initiation of the activator, the stopper must be properly siliconized.

##### Dual Chamber Syringes and Cartridges

From the experiences of one company within LyoHub, dual-chamber syringes and dual-chamber cartridges have very similar designs (cake and diluent are separated by a middle plunger stopper) and differ only in size, with DCSs being larger (up to 4 mL of diluent). For administration, a DCS needs only a needle and a plunger while a DCC requires a separate device. DCCs are typically placed within the device while DCSs are stored as a kit containing all the accessories needed for administration. The sequence of operations during the manufacturing of DCSs and DCCs is slightly different as compared to DCVs. First, syringes or cartridges are siliconized followed by the middle stopper placement. A middle stopper is positioned just below the bypass. Then devices are filled with the solution over the top of a middle stopper. Few dual-chamber containers have specially designed lyo stoppers placed after the filling operation in a semi-stoppered position allowing water to escape during lyophilization. During drying, DCSs/DCCs are typically positioned in a “tip-up” configuration where the cakes sit on the top of the middle plunger. After lyophilization of the semi-stoppered DCSs/DCCs, the shelves are collapsed (usually when the chamber is equilibrated at atmospheric pressure) sealing the drug product compartment. The DCSs/DCCs are then unloaded, turned upside down (typically by robotic systems), filled with the diluent, and sealed with the second stopper. The requirements for room humidity and environmental controls are drastically reduced. Some DCCs, however, are sealed outside of the drying chamber, so requirements for environmental control are the same as for a DCV or even tighter (RH < 10%).

During the administration of the drug product that is filled into the DCSs/DCCs, a plunger rod attached to the second stopper is used to press the stopper and move the diluent toward the middle stopper. The middle stopper is then moved, opening a bypass for delivering the diluent to the drug product compartment. After complete reconstitution, the solution can be directly administered to the patient; no additional steps are needed.

##### Trays

Tray drying is more common for the manufacture of active pharmaceutical ingredients (APIs) or their intermediates. The requirements for sterile manufacturing of these materials are not as strict as the manufacture of the parenteral drug product in vials or DCSs/DCCs as sterile filtrations will be carried out later when the API/Drug Substance is processed into Drug Product. Yet, the liquid product is typically filled into trays using principles of the aseptic technique to maintain a low bioburden. Trays may be covered during the transfer from the filling line to the shelves of the dryer. Trays are placed on the shelf, their cover is removed, and the operation is repeated until the dryer is fully loaded. To minimize the risk of cross-contamination and reduce product blow-out, disposable trays with water-permeable membranes are available commercially (21). Regardless of the tray design, lyophilized API or product intermediate needs to be transferred to the storage containers or sealed inside aluminum secondary containers to prevent moisture uptake.

Despite apparent differences in containers, the validation principles remain the same: demonstrate product uniformity and the ability to consistently manufacture products (8).

#### Specifics of Heat and Mass Transfer in Dual Chamber Devices

To understand better validation requirements or validation strategies for the dual-chamber containers, the specifics of heat and mass transfer of such containers need to be understood. The bottom of a DCV is essentially the same as the bottom of a typical tubing vial of the same diameter delivering essentially the same heat per unit of surface. The contribution from the top shelf may be slightly different since a stopper is not used during drying with a DCV, but the difference in overall vial heat transfer coefficient seems to be very small (unpublished data). It is worth noting that the resistance of semi-inserted stopper, which is used in regular vial freeze-drying, is not that significant compared to the dry cake resistance. Thus, the overall heat and mass transfer during lyophilization of a DCV is not that different from the regular vial freeze-drying process. Simple steady-state primary drying models (16) can be successfully used to design a lyophilization cycle for DCVs.

The heat transfer in dual chamber syringes and cartridges is not well understood compared to the vials. One of the reasons is that DCSs/DCCs need some sort of support (holders) during drying which is not standardized across the industry. The three-dimensional design and material of construction of this DCS/DCC support systems will significantly impact the heat transfer and the drying characteristics of the product. Few studies on the mechanism of heat transfer in dual-chamber containers have been performed (17–20). Patel and Pikal (17) explored the difference in the heat and mass transfer between custom made plexiglass and aluminum block holders showing significant improvement of heat transfer for aluminum block versus plexiglass holders (at 100 mT chamber pressure, the heat transfer coefficient is 1.7 times higher for aluminum block holders). They also reported there was almost no impact of pressure on *K*_v_ of plexiglass holders with an edge effect of about 70%. For the aluminum holder, the edge effect was only about 4% while the pressure effect on *K*_v_ was similar to that of a typical vial. Korpus *et al*. (20) investigated *K*_v_ as a function of pressure for a custom-made aluminum holder in which the cake was “suspended” above the holder body by about 10–20 mm. It was found that this configuration produced poor heat transfer compared to the regular vials. They also demonstrated a significant edge effect (50% more heat for the edge syringe at a pressure of 150 mT). In other publications, Korpus *et al*. (18, 19) explored different designs of holders: (1) aluminum holder (20); (2) “shell holder”, where each syringe is placed in the individual metal holder; (3) flexible holder, where part of syringes containing cakes was “immersed” in the massive aluminum block suspended above the shelf; (4) guide rail holder, where syringes were tightly packed within a metallic box while touching the shelf. The authors of these articles showed that the shell holder device was the most efficient for the heat transfer, both during freezing and drying, while also significantly reducing edge effects (18). However, overall energy transfer was still around three times less effective than that for freeze-drying in a traditional vial container system in the same pressure range. Also, cleaning of such holders may be a challenging task. Shell-type holders are commercially available and are called “puck systems” and are currently utilized by Boehringer Ingelheim (21).

Besides “needle up” systems, described above, “needle-down” technology was recently introduced by Werk *et al*. (22). As opposed to “needle up” systems, in which containers are held during drying with injection’s side up and then need to be inverted for the diluent filling, the container during the “needle-down” process (drug product filling, drying, and diluent filling) remains in the same orientation throughout. This allows keeping product close to the shelf and, with the implementation of advanced holders (aluminum blocks), guarantees good heat transfer. The potential risk of using such systems could be incomplete reconstitution of dried product residues in Luer cone or staked-in needle configurations and challenges during injection as a result of potential needle plugging.

**Fig. 4:**
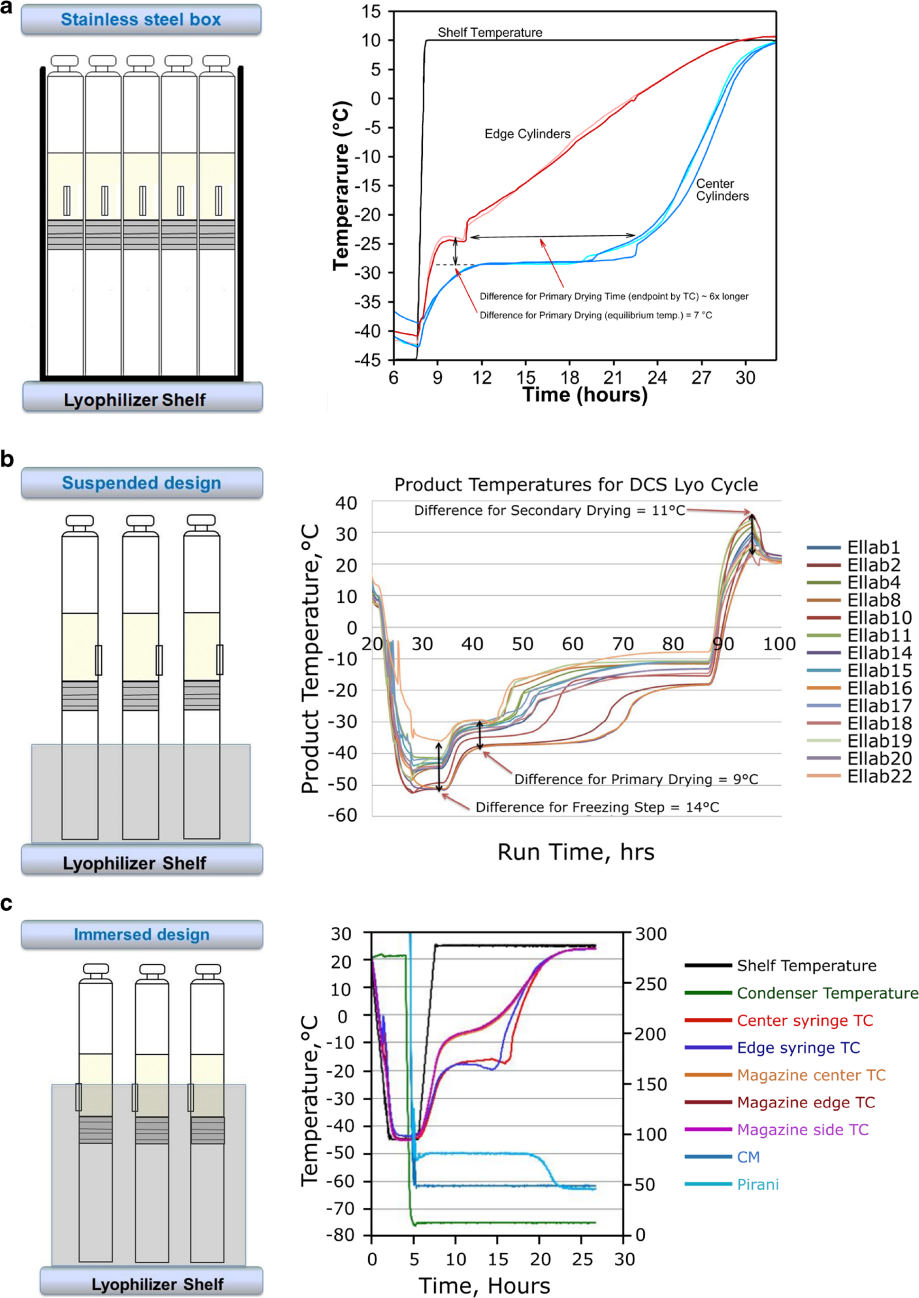
Evolution of dual-chamber holders used in commercial manufacturing of biopharmaceutics. The three panels represent the different designs of holders (inset left) and temperature traces for edge and center syringes (inset right). **a** Position of DCSs that are tightly packed in a stainless box. **b** The most commonly used syringe holder (“suspended design”). **c** Newly designed holders where cakes are in close contact with the block (“immersed design”)

An example evolution of commercially manufactured drug products in dual-chamber systems is shown in Fig. 4. In the original configuration, syringes were closely packed inside a metal box (Fig. 4a). Boxes were manually loaded on the shelves of the freeze-dryer. Temperature traces from the lyophilization process in this configuration show a significant temperature gradient (approximately 7 °C) between edge and center syringes. The apparent drying time, indicated by the temperature sensor, was almost 6 times longer for the center syringe. These data are comparable to the guiderail systems discussed by Korpus *et al*. (18). Poor heat transfer and significant edge effect necessitated design changes of the holders (“magazines”) where syringes were separated to minimize the “shielding” effect (23). To improve heat transfer, new holders were made of an aluminum alloy. Likely to reduce the weight of the “magazines”, holders were made short, leaving cakes above the heat transfer surface and allowing significant radiation from the walls of the dryer (“suspended design”—Fig. 4b) (24). Edge effects caused a substantial temperature gradient between edge and center containers during both freezing (14 °C) and drying (9 °C). Therefore, this resulted in some variability of product quality (differences between center and edge syringes in the degree of crystallinity, fragility, and *T*_g_ of dried material). These differences required significant validation efforts to prove the comparability of the product within the batch. With a “suspended design”, DCCs had a similar edge effect as compared to the DCSs. The design of the holders was further improved so that DCCs were, at least partially, covered by the “magazine” body; the so-called “immersed design” (Fig. 4c) (24). This “immersed design” resulted in close contact of the DCS with the holder resulting in improved heat transfer and significant reduction of the edge effect. The difference between edge and center containers was within 1 °C during freezing and earlier stages of drying (Fig. 4c). Due to improved homogeneity, an acceptable product temperature design space could be notably narrowed resulting in a less extensive robustness study.

**Fig. 5:**
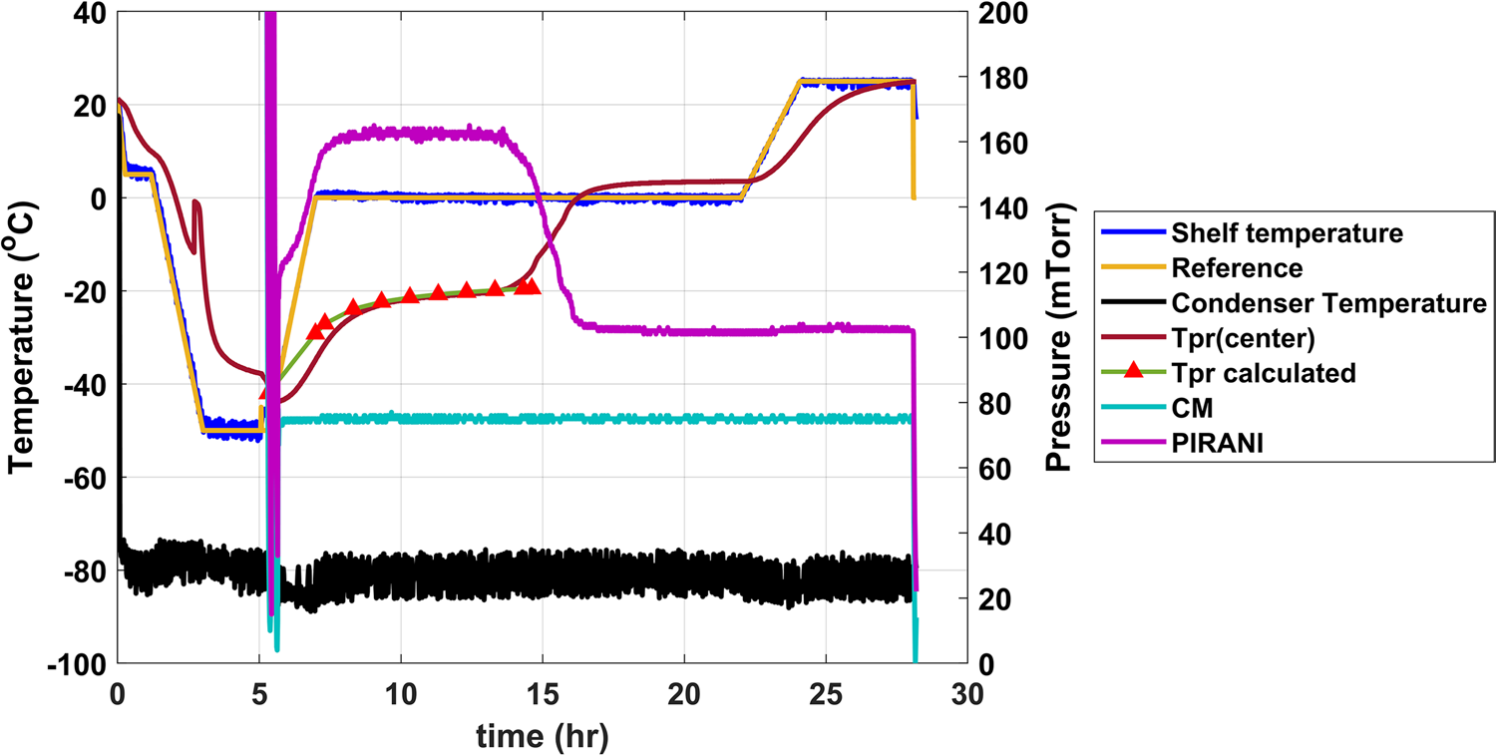
Comparison of calculated (red) and actual product temperatures for DCSs, freeze-dried in a “suspended design” holder. A steady-state model (12) of primary drying was used in calculations. Formulation consisted of 200 mg/mL protein, 5% sucrose and 10 mM histidine

Unlike drying in vials, a robust primary drying model for DCSs/DCCs has not been developed yet. For the “suspended design” holder type, a steady-state primary drying model was successfully used to predict the product temperature profile (Fig. 5) (24). In this model, the contact area between the product and glass was considered as a heat transfer surface. For the “immersed design” holder, the contribution of the non-steady-state part of the cycle (~ 12% of total heat utilized during sublimation (24)) may not be completely neglected.

#### Specifics of Heat and Mass Transfer in Tray Drying

Freeze-drying in trays is a well-established technology used in food and pharmaceutical industries to process in bulk. It is therefore not surprising that robust primary and secondary drying models were developed more than 20 years ago (25). In the biopharmaceutical industry, to the authors’ knowledge, tray drying is mostly used for the manufacture of drug substance, drug product intermediates, or APIs. Tray drying is generally conducted either in disposable plastic or reusable metal trays. Freeze-drying in trays has some specific considerations compared to vial freeze-drying. For example, solidification of water, after initial supercooling and ice nucleation, takes longer in trays versus vials. In some cases, one can visually see a movement of the freezing front from the edges to the center of the tray. In contrast, the freezing front progresses through the vial almost instantaneously. Due to slower freezing in trays, one could expect a larger cryo-concentration effect as compared to the vials and a higher likelihood of skin formation. Additionally, shrinkage and cracking of the product may be more pronounced in tray drying compared to vial drying for the same product. This could result in a reduction of heat transfer towards the end of primary drying. As an example, lyophilization in trays was compared to vial freeze-drying using a 5% sucrose solution as a model material. While the heat transfer coefficient of the vial and the tray at 50 mTorr were determined to be comparable, it took at least 20% longer for tray drying as compared to vial freeze-drying. Significant cracking and potential loss of contact with the bottom of the tray was suspected as a reason for the increase in drying time.

Reusable metal trays may be more robust to tearing and scratching when compared to plastic trays, but they may warp with time due to thermal treatment such as sterilization or washing which could change the tray heat transfer coefficient. Variability in heat transfer from shelf to tray (warping) or from tray to product (cracking) could significantly influence the endpoints of both primary and secondary drying. The endpoint determination (by pressure comparative method or pressure rise test) is more important for tray drying as compared to the vial drying, where vials are in direct contact with the shelf.

#### Validation Approaches for Dual Chamber Devices and Tray Drying

The validation strategy for products lyophilized in DCVs is similar to that of regular vial freeze-drying. Sterile filtration, filling, loading, and drying processes are almost the same for both media. One should also expect a similar product temperature design space because the heat transfer is not that different as compared to the regular vial freeze-drying. The biggest difference is that DCVs are not stoppered inside the dryer. The additional focus of the validation procedure for a DCV, therefore, is to demonstrate that product remains sterile and particle-free during unloading of a DCV and before the placement of the middle stopper. Also, moisture uptake before stopper placement should remain within the target, which is typically much lower than the upper limit of specification acceptance criteria. Maximum hold times and relative humidity limits should be defined between the unloading of the lyophilizer and the closure of the last container.

In DCS/DCC freeze-drying, due to poor heat transfer, metal boxes are not currently used. Therefore, the authors of this paper will focus on the validation of processes in “suspended design” and “immersed design” holders. In the example of”suspended design” (Fig. 4b), the lowest product temperature for an edge syringe was about – 36 °C while the shelf inlet temperature set point varied between – 52 °C and – 56 °C (lowest achievable set point for this dryer). This means that if the product required temperatures below – 36 °C during freezing, this may not have been achievable for a portion of the batch in this type of holder for this particular dryer. Also, the difference in temperature between edge and center syringes was about 9 °C and 11 °C during primary and secondary drying, respectively. As a result, substantial robustness studies are needed to support a wide drug product temperature design space. Since the edge effect strongly depends on wall temperature, the best validation practice should also include the monitoring of the wall temperature during engineering runs to make sure that the edge effect is reproducible and acceptable for the edge containers. A minimum process restriction between the heat sterilization of the lyophilizer and the start of the lyophilization process needs to be in place to avoid unacceptable temperature distributions within the product. Ideally, modern freeze-dryers could be built with control and monitoring systems for the doors and walls. The impact of the process parameters’ variability on product quality should be documented, at least on a laboratory scale, to demonstrate the robustness of the commercial process. Freeze-dryers should also be capable of reducing the product temperature below the target for every single container during freezing regardless of the position. This may require validation of equipment capability and continued process verification to reach that low temperature and demonstrate that temperature is achievable across the shelf and between the shelves as a function of load. For example, for products with *T*_g_' < – 40 °C, shelf temperature should be below – 60 °C for “suspended design” holders (Fig. 4b). If the formulation contains a bulking agent that requires a low nucleation temperature (mannitol, sodium chloride), a temperature of – 36 °C may not be low enough to ensure complete crystallization. Variability in the crystallinity of the bulking agent could therefore be expected for the materials freeze-dried in this type of container holders. In addition to typical CQAs measured during PPQ (moisture, reconstitution time, other product specific QAs), the degree of bulking agent crystallinity, the specific surface area of dry powder, and the Tg may be required to ensure that the quality of drug product produced during PPQ material remains within proven design space. Also, a substantial sampling plan (i.e., few locations from each shelf within the chamber) at PPQ is required to prove the homogeneity of the product during lyophilization.

Figure 4c depicts excellent heat transfer for the “immersed design” holders. The difference between edge and center DCCs did not exceed 1 °C until the later portion of primary drying. Unlike the notable differences between edge and center in the “suspended design”, the temperature gradient during secondary drying was less than 1 °C. The edge effect was even lower than that during typical vial drying. Thus, a robustness study and validation efforts for the “immersed design” configuration could be much less intensive, including a reduced sampling plan (i.e., few locations from the bottom, middle, and top shelves of the chamber). Variability in product quality may also be minimal due to very narrow design space and non-typical testing (degree of crystallinity, for example) may not be required. Some DCCs are not semi-stoppered before loading into the dryer and after completion of the cycle, lyophilized powder is exposed to environmental conditions until the last DCC is sealed. Thus, similar to DCVs, validation exercises should demonstrate that the product remains sterile and particle-free with the moisture being within specification during unloading of DCCs.

A few challenges in the validation of products in dual-chamber containers are related to specifics of its design. Unlike with the regular vial configuration, in dual-chamber containers, drug product, diluent, or both are always in contact with the stopper. In most cases, to function properly after reconstitution, stoppers and glass barrels need to be siliconized before the filling and drying. Siliconization of stoppers could impact the number of leachables during storage which has to be evaluated concerning the product quality. Silicone oil could interfere with assays, so alternative analytical methods or more efforts in assay validation may be required for the dual chamber configurations. Siliconization could also impact product quality (particle formation in reconstituted solution as one such example), so additional robustness study may be required with representative containers. It is good practice for a robustness study to be performed with the containers treated the same way as would be expected during commercial manufacturing (depyrogenation, siliconization, etc.). One could also expect moisture transfer through the stopper during long-term storage (15). Thus, the stability of the product, associated with moisture transfer and leachables uptake, could vary depending on the commercial process. This could delay product approval from regulatory agencies as they may require actual stability data from a particular commercial site. Also, the strength of a dual-chamber product can be based on both the filling accuracy of the active and diluent fill (depending on how the strength method is designed). This combined with the low fill weight in DCC puts a high demand on the accuracy of the filling line.

## CASE STUDIES

The process validation is exemplified with three case studies which include the validation strategy for different filling volumes and equipment; the chamber loading process effect on product quality and special case of the product in DCC lyophilization. The details of each case study are provided below.

### Monoclonal Antibody Case: Fill Volumes and Equipment Validation Strategy

A commercial monoclonal antibody lyophilized product with two different vial strengths (‘a’ and ‘b’ mg/vial) is to be transferred within the same commercial manufacturing site to a different suite, resulting in a change of filling line and lyophilizer equipment. Besides, the new suite has two different lyophilizers (Lyo A and Lyo B) that would be used to manufacture the product. The PPQ protocol in the new suite consisted of four runs in total with two batches per product strength: three validation batches to be manufactured in one lyophilizer (Lyo A) to demonstrate batch-to-batch consistency and a fourth validation batch to be manufactured in the second lyophilizer (Lyo B) to demonstrate the equivalent performance of the two lyophilizers. The protocol requires that lyophilization process validation should be conducted under anticipated commercial manufacturing conditions. Performance qualification of critical processing equipment was conducted to verify that the equipment performed consistently within defined operating parameters when subjected to normal production conditions. Heat and moisture mapping studies were performed to demonstrate that the two lyophilizers were functionally equivalent. The operational process steps (e.g., filling, lyophilization) for the manufacturing of both strengths were similar, and the vial size and composition were the same for both product strengths; the only difference was in their respective filling volumes.

Table XII shows the validation strategy included in the PPQ protocol at the new suite. Lot 1 represents the worst-case lyophilization condition for condenser capacity as vial strength ‘b’ had a higher fill volume, i.e., a higher amount of water content (per vial) to be sublimed during primary drying. Lot 3 represents the best-case lyophilization condition, as vial strength ‘a’ had a lower fill volume. Lyophilization-related parameters such as shelf temperature and chamber pressure during drying are considered to be critical process parameters and were monitored in-process. The normal operating range was established by trending process performance from development batches and equipment capability and was to be verified during validation. Samples are to be collected from locations throughout the lyophilization chamber for each batch size (top-middle-bottom, front-middle-back, alternate, even, and odd shelves, the worst-case location from shelf mapping) after the cycle. This sampling plan was proposed to demonstrate that products from all locations met pre-determined quality attributes. Testing verification with respect to the lyophilization process will be performed on the finished goods. The quality attributes of the lyophilized drug product will be demonstrated by analytical tests (e.g., moisture content, cake appearance, product reconstitution time, degradants, particulates, potency) and a microbial test (BET). This process validation strategy is expected to demonstrate that the manufacturing process was robust, capable, and reproducible at the target, upper, and lower limits of the proposed variable batch size (110 L–250 L) and that all CQAs and specifications associated with both product strengths are robust and reproducible at the new suite.

**Table XII:** Process Validation Matrix

Lot	Batch size	Vial strength (mg/vial)	Lyophilizer
1	250 L	b	Lyo A
2	110 L	b	Lyo A
3	110 L	a	Lyo A
4	200 L	a	Lyo B

### Impact of Lyophilization Chamber Loading Process on Product Appearance and Product Rejection Rates

Process design, process qualification, and continued process verification ensure the development and scale-up of a robust, reproducible commercial manufacturing process while providing ongoing assurance that the process remains in a state of control. The case study is shown here, however, documents user experience associated with lyophilization of a complex live virus vaccine formulation. The reader is reminded that vaccines, by their intrinsic instability, often requires the investigator to define additional CPPs such as Time in Solution (TIS), Time out of Refrigeration (TOR), etc., and in many instances are sensitive not only to the freezing protocol but also on final moisture content. Flash freezing, for example, is the freezing method of choice for live virus vaccines (LVVs) to minimize TIS. Similarly, low moisture may often result in low-drying yields for an LVV while too high a moisture may compromise the shelf-life of the virus, and thus an optimal moisture condition is empirically determined. This is further illustrated by the live virus vaccine formulation case study wherein the chamber loading process had a significant effect on cake appearance due to inadvertent annealing during the loading step. The reader is referred to Wallen *et al*. (26) for a detailed description of the study design and a brief description of the study is provided below.

A validated freeze-dryer consisting of 18 mobile shelves with an out-swing door was used for the study. The form/fill process consisted of filling < 1 ml (fill height of 4.2 ± 0.2 mm) of the formulation components in a 3-cc type 1 glass tubing glass vials partially stoppered with a slotted butyl rubber stopper. Vials were placed on a perforated tray and the product was immediately flash-frozen to a temperature of approximately – 100 °C using direct liquid nitrogen (LN2) injection freezer. Note that this process was optimized for the LN2 freezer to accommodate the sensitivity of the complex live virus vaccine. The product, once flash-frozen, was loaded onto freeze-dryer shelves that were cooled down to – 50 °C manually by opening the dryer door, placing several trays (8 trays/shelf with 4 trays in the front and 4 in the back), and closing the door. This process was repeated to fill the dryer, and the lyophilization cycle was initiated post final door sealing once all trays were loaded. It was observed that the loading method impacted the rate of product collapse during the freeze-drying process. Inadvertent annealing resulting from indexing of shelves during the loading process resulted in product collapse. This is further described in greater details below while results (shows as percentage of vial collapsed) is described in Table XIII.

Engineering batches in the production settings were successfully executed utilizing the fully expanded shelf configuration (referred to as fully indexed shelves) thereby indicating the ability to utilize the full dryer without any product impact. While transitioning to a new dryer within the same facility, an attempt was made to optimize the loading process by indexing the shelves during the loading process. This was achieved by compressing the shelves at the start of the loading process followed by the sequential raising of several shelves and loading the product on the exposed shelves. In contrast to loading on fully indexed shelves, indexing during loading resulted in a marked increase in the rate of product collapse observed resulting in an increased rejection rate (Table XIII). A root cause investigation determined the main cause of collapse be associated with inadvertent annealing and, in certain cases, product exceeding *T*_g_’ when shelves were indexed during the loading process. This was attributed to the fact that shelves when compressed have a lower exposed surface area and corresponding capacity to rapidly chill the cold air entering the chamber and created air circulation during indexing. In contrast, fully expanded shelves act as a heat sink to remove heat from incoming warm air due to a much larger surface area. A comparison and contrast between the CQAs (moisture, reconstitution time, and potency) revealed similar potency and reconstitution time between elegant and collapsed product; however, the moisture was approximately twofold higher in the collapsed product. As a remediation approach, usage of the top few shelves of the chamber was discontinued.

**Table XIII:** Product Rejection Rates as a Function of Lyophilization Chamber Loading Process. Please Note Shelf 1–4 were not utilized in the study

Chamber shelf #	Rejection rate as a function of indexing during loading	Rejection rate as a function of fully indexed shelf
5–6	20.0%	0.2%
7–8	13.2%	0.4%
9–15	0.8%	0.9%
16–18	Empty	0.2%

Understanding the process transfer between cabinets, as documented in the case study above, is therefore critical and relies on the fundamental understanding of the formulation and the process science as unwanted annealing may impact product quality. Thus, the authors recommend that proper temperature mapping studies must be performed between equipment and facilities to ensure proper and efficient technology transfer.

### Lyophilization Cycle for a Product in a Dual Chamber Cartridge

**Fig. 6:**
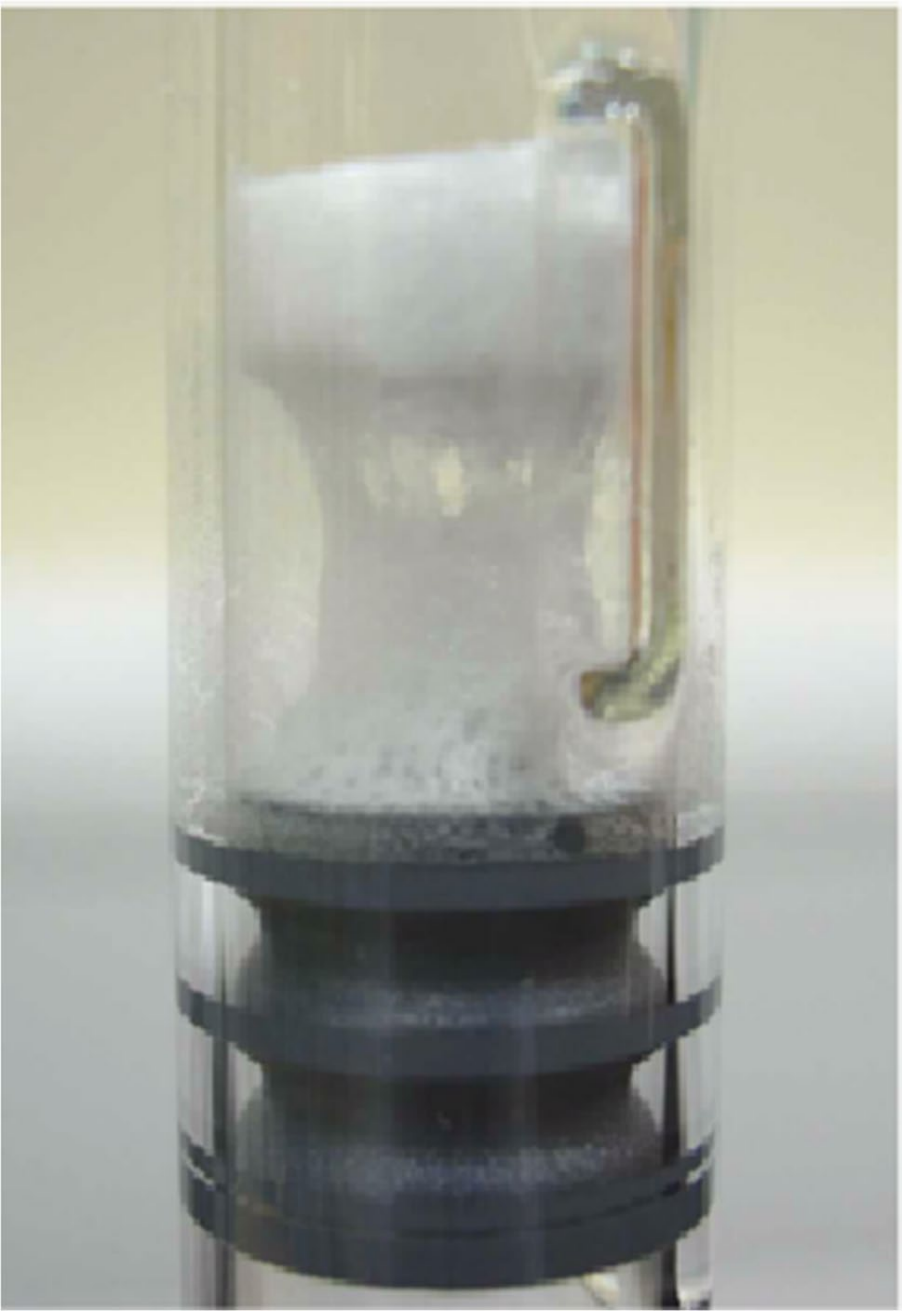
A Sample of a Collapsed Cake occurring in DCCs

Freeze-drying in Dual Chamber Cartridge (DCC) serves as one approach to improve patient compliance when coupled with a pen injector device thebery enabling self-administration. DCC fills minimizes overfill, increase dose accuracy while provding ease of reconstitution; however, the freeze-drying process itself is more complex than the vial drying process. Considerations for optimizing heat and mass flow in DCC are well documented in literature showcasing impact of process parameters on product CQAs (16–22). Additionally, specifics of heat and mass transfer in dual chamber containers is described in “Dual chamber vials” section. This case study here focuses on a sucrose-based formulation with a *T*_c_ of – 31 °C in a DCC with the product to be lyophilized sitting on an insulator (rubber stopper) 4 cm above the shelf surface. Specifically, lessons from drying the product in a DCC, which contrasts with the vial drying process, are documented here with an illusttrative example of a collapsed product in DCC. It should be noted that heat transfer in such a scenario may be convection (or radiation but not conduction) dominated and horizontal through the sidewalls as opposed to the bottom of a vial. Furthermore, sublimation through the small diameter cartridge tip creates a flow restriction that may further complicate the drying process. Also, the edge effect may be substantially different under these conditions. Additional heat from the chamber walls that are insulated may cause cartridges at the shelf edges, particularly the corners of the top and bottom shelves, to dry significantly faster than the rest of the shelf causing lidded cakes (initial sublimation with retention followed by a section of total collapse; a collapse within a DCC is portrayed in Fig. 6). The product can sublime at a relatively high temperature, but shrinkage occurs as the product temperature spikes at the end of sublimation. Shrinkage with horizontal heat transfer results in an hourglass cake. Both collapse and shrinkage increase residual moisture which, in turn, may negatively impact stability especially under scenarios like the one tested here wherein the primary degradation pathway is hydrolysis.

To assure all cartridges are within the moisture specification and produce a uniform cake appearance, each cartridge must end sublimation at essentially the same product temperature. This was achieved by utilizing high heat transfer (high shelf temperature and chamber pressure) at the start of sublimation to warm edge cartridges quickly to optimal sublimation followed by slowing heat transfer to a minimum as the corners of the shelves end sublimation. The edge cartridges were monitored and once edge cartridges end sublimation, all remaining cartridges were observed to be drying sub-optimally. The heat transfer was then increased gradually to chase the end of sublimation from the corners to the middle of the shelves such that all cartridges end sublimation at a similar temperature to achieve an efficient drying cycle.

## SUMMARY AND FUTURE OUTLOOK

This paper is the second of two-parts that describe best practices in the industry for lyophilization process design and validation. In the first part (Part I: Process Design and Modeling), recommended best practices in lyophilization process design are discussed in detail. The second part focuses on the best practices for the PPQ and CPV stages of the validation of the lyophilization process. Here, we provide a critical update to previously published literature on this topic leveraging insights from multiple biopharmaceutical companies, including guidance for equipment operational qualification, PPQ, and CPV.

Specifically, best practices for batch size determination were discussed, including the impact of batch size on drying time, careful selection of process parameters to avoid product failure and to support lyophilization of the maximum batch size as a worst-case scenario. Also, batch size overage to compensate for losses during production were considered. Sampling strategies to demonstrate batch uniformity were also discussed, including sampling from worst-case locations, leveraging prior knowledge of shelf-temperature variability and relevant CQAs. The use of statistical models to ensure adequate sampling to account for variability and the probability of failing specifications are considered.

Best practices for determining the number of PPQ runs for various scenarios were presented through a survey of LyoHUB member organizations. The recommendations are centered on a bracketing approach considering maximum and minimum lyophilizer loads. Additionally, standard practice around CQA and CPP selection was outlined, and the benefits of using control charts and run charts for process trending and quality control were described, in addition to methods used to plot data in these charts. Finally, validation approaches were illustrated through case studies that covered (i) the validation strategy for a monoclonal antibody with 2 strengths lyophilized in 2 freeze-dryers, (ii) the impact of the loading process on the lyophilization cycle and product quality for a live virus vaccine, and (iii) the design of a lyophilization cycle for a dual chamber cartridge system.

In addition to the standard practices in the validation of the lyophilization process, special lyophilization processes and the impact thereof on the validation strategy have been discussed in this paper. Nevertheless, the knowledge and experience to scale-up of this technology for commercial manufacturing remains rudimentary. As such, development work and manufacturing experience are required to identify and characterize CPP that are specific to this technology, and to select the appropriate approach to their evaluation during the manufacturing process validation campaign.

Another example of special cases is the lyophilization in alternate primary packaging systems such as dual chamber vials, syringes, and cartridges. As the number of products with such presentations is small, commercial manufacturing experience is limited. Accordingly, the validation of such lyophilization processes should take into account heat- and mass transfer differences between plexiglass and aluminum holders, holder design (‘suspended’ and ‘immersed’), the differences between ‘needle-up’ and ‘needle-down’ systems, and the potential impact of siliconized stoppers on product quality and stability. In this vein, recommended best practices for alternate lyophilization container-closure systems include (i) the monitoring lyophilizer wall temperature to better understand edge effects, (ii) ensuring adequate down-time between lyophilizer sterilization and start of freeze-drying to minimize impact of shelf temperature variability, (iii) using shelf temperatures sufficiently lower than the product *T*g’ for process homogeneity, and (iii) running a robustness study or engineering batch using the same process parameters, equipment, and packaging components as those intended for commercial manufacturing.

For the most part, lyophilization process scale-up and validation has been based on prior experiences and conventional scale-up factors and bracketing approaches. Over the past two decades, modeling of the primary drying phase and of the equipment capabilities have been significantly advanced. Nevertheless, most modeling efforts are still limited to the process design stage and to some extent to process scale-up and technology transfer. The potential use of modeling to guide the design of the validation protocol of the lyophilization process is still lacking. More work on the parts of the manufacturers as well regulators is required in order to formalize the use of process modeling in validation campaigns and in regulatory filings.
